# Integrated Network Pharmacology Approach for Drug Combination Discovery: A Multi-Cancer Case Study

**DOI:** 10.3390/cancers14082043

**Published:** 2022-04-18

**Authors:** Antonio Federico, Michele Fratello, Giovanni Scala, Lena Möbus, Alisa Pavel, Giusy del Giudice, Michele Ceccarelli, Valerio Costa, Alfredo Ciccodicola, Vittorio Fortino, Angela Serra, Dario Greco

**Affiliations:** 1 Finnish Hub for Development and Validation of Integrated Approaches (FHAIVE), Tampere University, 33100 Tampere, Finland; antonio.federico@tuni.fi (A.F.); michele.fratello@tuni.fi (M.F.); lena.mobus@tuni.fi (L.M.); alisa.pavel@tuni.fi (A.P.); giusy.delgiudice@tuni.fi (G.d.G.); angela.serra@tuni.fi (A.S.); 2 Faculty of Medicine and Health Technology, Tampere University, 33100 Tampere, Finland; 3 Department of Biology, University of Naples “Federico II”, 80138 Naples, Italy; giovanni.scala@unina.it; 4 Department of Electrical Engineering and Information Technology (DIETI), University of Naples “Federico II”, 80138 Naples, Italy; michele.ceccarelli@unina.it; 5 Institute of Genetics and Biophysics “Adriano Buzzati-Traverso”, CNR, Via P. Castellino 111, 80131 Naples, Italy; valerio.costa@igb.cnr.it (V.C.); alfredo.ciccodicola@igb.cnr.it (A.C.); 6 Department of Science and Technology, University of Naples “Parthenope”, 80143 Naples, Italy; 7 School of Medicine, Institute of Biomedicine, University of Eastern Finland, 70211 Kuopio, Finland; vittorio.fortino@uef.fi; 8 Institute of Biotechnology, University of Helsinki, 00100 Helsinki, Finland

**Keywords:** druggability, network, systems pharmacology, cancer, cancer therapy, drug repositioning, drug combinations

## Abstract

**Simple Summary:**

Current treatments for complex diseases, including cancer, are generally characterized by high toxicity due to their low selectivity for target cells. Moreover, patients often develop drug resistance, hence becoming less sensitive to the therapy. For this reason, novel, improved, and more specific pharmacological therapies are needed. The high cost and the time required to develop new drugs poses the attention on the development of computational methods for drug repositioning and combination therapy prediction. In this study, we developed an integrated network pharmacology framework that combines mechanistic and chemocentric approaches in order to predict potential drug combinations for cancer therapy. We applied our paradigm in five cancer types, which we used as case studies. Our strategy can be applied to the study of any complex disease by guiding the prioritization of drug combinations.

**Abstract:**

Despite remarkable efforts of computational and predictive pharmacology to improve therapeutic strategies for complex diseases, only in a few cases have the predictions been eventually employed in the clinics. One of the reasons behind this drawback is that current predictive approaches are based only on the integration of molecular perturbation of a certain disease with drug sensitivity signatures, neglecting intrinsic properties of the drugs. Here we integrate mechanistic and chemocentric approaches to drug repositioning by developing an innovative network pharmacology strategy. We developed a multilayer network-based computational framework integrating perturbational signatures of the disease as well as intrinsic characteristics of the drugs, such as their mechanism of action and chemical structure. We present five case studies carried out on public data from The Cancer Genome Atlas, including invasive breast cancer, colon adenocarcinoma, lung squamous cell carcinoma, hepatocellular carcinoma and prostate adenocarcinoma. Our results highlight paclitaxel as a suitable drug for combination therapy for many of the considered cancer types. In addition, several non-cancer-related genes representing unusual drug targets were identified as potential candidates for pharmacological treatment of cancer.

## 1. Introduction

Drug repurposing is currently a research mainstream in predictive pharmacology. Its diffusion is due to the significant need for novel strategies aimed at safely bringing drugs to market more rapidly and cost-effectively [1]. A successful drug repurposing application allows the bypassing of the burden of de novo drug development, including safety assessment and regulatory procedures, providing innovative therapeutic protocols for a plethora of pathological conditions [2].

Mechanistic drug repositioning relies on pattern matching approaches, under the assumption that an effective drug is capable of reverting the molecular alterations of a specific disease. This approach is referred to as “connectivity mapping”, and it was first suggested by Lamb and collaborators in 2006 [3]. Likewise, chemocentric approaches to drug repositioning are especially limited to structural and docking-related investigations, following the assumption that an effective drug is capable of physically interacting, and possibly changing the function of specific targets. In the context of mechanistic drug repositioning, network pharmacology approaches have shown to be a precious instrument that accelerates the discovery of novel associations between drugs and diseases [4]. These advances have raised the possibility of moving beyond the “one disease-one drug” paradigm, exploring the “one disease-multiple drugs” dimension, which can significantly ameliorate the therapeutic management of complex diseases [5]. A long-known problem of multi-drug interactions is the assessment of the combinatorial effect of a simultaneous treatment of multiple drugs on a biological system. Network-based representations of gene–gene co-expression interactions greatly enhanced the prediction of such synergistic effects on a large scale, in contrast with the experimental evaluation of single drug–drug interactions. Network pharmacology approaches simultaneously evaluate the molecular impairment of the disease under study and the mechanism of action of drugs, underlying network targets for a certain disease [6]. On the other hand, the use of model-based predictive approaches offers advantages, for example, it is possible to directly predict the efficacy of a drug combination (e.g., direct prediction of the IC50) [7]. This is in contrast to network-based models, where it is possible to prioritize drug combinations based on topological and molecular properties [7,8]. However, the use of machine learning (ML) comes with its own difficulties and limitations. ML models are data hungry: the amounts of data required to properly fit ML models grow exponentially with complexity, which is one of the major issues in the context of drug synergy prediction as data acquisition is extremely costly, both in terms of time and resources, though this issue is somehow ameliorated by the availability of publicly available datasets [9,10,11,12]. Feature extraction is challenging; therefore, a lot of effort has to be invested in constructing meaningful features. For example, Ji et al. developed a gradient boosting machine trained on five features based on pharmaceutical, phenotypic and mechanistic properties that encode the differences between two given drugs to predict their chance of exerting a synergistic or agonistic effect [13]; ML models (especially the most complex ones) are prone to fit the training data too well and achieving reduced capabilities to generalize to new data (overfitting). In order to avoid this, proper validation, model selection and inspection methodologies must be ensured.

Nonetheless, the exploration of the topological configurations of proteins involved in a disease and the targets of candidate drug combinations provides precious insights about what constitutes a good candidate combination [6]. Notably, Cheng et al. systematically analyzed all the possible configurations of the targets of a combination of two drugs and the proteins relevant to a disease embedded in a PPI network and identified that the probability of a drug combination to have a synergistic effect is higher when (1) both drugs need to target proteins in the disease network and (2) the drugs should share as few as possible targets. As a matter of fact, the number of shared targets between the drugs in the combination is positively correlated with the presence of adverse outcomes [5].

However, the current strategies solely exploit the counterbalancing of a certain drug on the perturbed expression in a disease state [2]. Such methods overlook the intrinsic characteristics of the drugs, such as their mechanism of action and chemical structure. We have already proved that the integration of the structural and the mechanism of action (MOA) properties of the drugs improves our ability to predict their therapeutic class [2]. Recently, we showed that hybrid quantitative structure–activity relationship (QSAR) modeling, comprising both structural and mechanism of action features, enhances the prediction of the affinity binding of drugs to human albumin [14,15] and proposed a computational strategy that combines multiple bioinformatics and cheminformatics methods to prioritize drugs for the treatment of COVID-19 disease [14,15].

In this work, we developed an integrated method aimed at the identification of drug combinations which takes into account both molecular characteristics of the disease under consideration (mechanistic approach) and the intrinsic characteristics of the drugs, such as their mechanism of action and chemical structure (chemocentric approach). Our method relies on the fact that (1) the repurposing is based on networks rather than on simple pattern matching between drug sensitivity and disease-related gene expression signatures, allowing a thorough characterization of the relationships among the deregulated genes; (2) our approach takes into consideration multiple aspects of drug–drug and drug–target interactions, including topological properties of target genes within the network, drugs’ mechanism of action and their chemical structure; (3) disease network models allow to evaluate the effect of drugs’ combination. To show the effectiveness of our method, we present five case studies, taking advantage of publicly available transcriptomics data.

## 2. Materials and Methods

### 2.1. Data Collection

All the analyses performed in this work were carried out using publicly available data. RNA-sequencing data of invasive breast cancer (BRCA), colon adenocarcinoma (COAD), hepatocellular carcinoma (LIHC), lung squamous cell carcinoma (LUSC) and prostate adenocarcinoma (PRAD), were retrieved from The Cancer Genome Atlas (TCGA), hosted at the Genomics Data Common portal (https://portal.gdc.cancer.gov (accessed on 6 November 2017)).

In order to achieve statistically robust results, we selected cancer types for which gene expression data for at least 10 matched samples (tumor sample and healthy counterpart) were available. Transcriptomics data were retrieved and downloaded through the use of the Bioconductor package TCGAbiolinks [16]. For gene expression analysis, we retrieved the UQUA-FPKM normalized HTSeq counts.

Drug-target associations were retrieved from the Open Targets database (https://www.opentargets.org (accessed on 15 January 2018)).

To establish the mechanism of action (MOA) of the mapped drugs, we retrieved from the L1000 repository the transcriptional profiles of all the drugs tested at the dose of 10 uM and time point 24 h in cell lines representative of the cancer under consideration (https://lincsproject.org (accessed on 19 January 2018); [17]). Specifically, we selected the following cell lines: MCF7 for BRCA, HEPG2 for LIHC, PC3 for PRAD, HT29 for COAD and A549 for LUSC. Since Subramanian and colleagues showed that a reduced representation of the transcriptome (constituted by 978 genes) recovers about 82% of the information of the entire transcriptome, we focused the analysis of L1000 signatures on such landmark genes. In order to infer a robust co-expression network to represent the MOA of included drugs we restricted the analysis only to those profiles for which more than five replicates were available.

Based on such restraints, our study includes 316 drugs for BRCA, 62 drugs for COAD, 87 drugs for LUSC, 11 drugs for LIHC and 294 drugs for PRAD, for a total of 430 unique drugs.

Furthermore, we retrieved the drug secondary structures as Simplified Molecular Input Line Entry System (SMILES) from the PubChem repository (https://pubchemdocs.ncbi.nlm.nih.gov/about (accessed on 15 January 2018)).

### 2.2. Gene Expression Analysis

In order to identify relevant candidate genes for the construction of the co-expression networks, we selected, in each cancer, two classes of genes: genes whose expression is significantly different between the tumor and the healthy counterpart, and genes whose expression is significantly more variable in one condition with respect to the other. The first class of genes was identified by performing a Wilcoxon signed-rank test [18] between tumor and healthy counterparts and the second class of genes was identified by an F-test on the variance between the two conditions. Notably, the *p*-values obtained from the two tests were not directly used as a filtering criterion. Rather, such *p*-values were used in order to rank the most deregulated genes by computing a significance score (SS) as follows:

SS^t^_i_ = abs(logFC_i_ × −log(*p* value^t^_i_)) (1)

where i represents the gene and t is either the Wilcoxon or F-test. The two lists of scores were ranked from the highest to the lowest value. The union of the genes within the top 10% of both ranks were selected for further analyses.

### 2.3. Co-Expression Networks Inference

Co-expression networks of both the cancer and the L1000 transcriptional profiles were inferred by using the INfORM algorithm [19]. INfORM relies on an ensemble method in order to infer robust gene co-expression networks. In detail, we ran INfORM by using all possible combinations among clr [20], ARACNE [21] and MRNET [22], all possible correlation and mutual information measures, and all of the discretization methods included in the minet R package [23]. INfORM gives in output the consensus of all the adjacency matrices built over all the combinations (for details, see [19]).

In order to identify the hub genes in each network, we ranked the nodes based on several centrality measures (degree, shortest path, betweenness, closeness, eigenvector) and the significance score (SS), and obtained a consensus through the use of the Borda function from the TopKLists package [24].

### 2.4. Construction of the Multi-Layer Network

Heterogeneity of publicly available data makes combined analysis often challenging and requires a significant amount of manual (pre-)processing. We proposed previously the Unified Knowledge Space (UKS) [25] as a platform to counteract the existing heterogeneity and integrate a wide range of available data types into a single data space. The UKS is a Knowledge Graph [26,27], containing different types of biological data, encoded in the form of nodes linked by edges (relationships).

For this study, physical protein–protein interactions, gene–gene regulation as well as functional associations were collected from multiple sources (Table S1). Due to technical reasons, proteins, transcripts and genes are mapped to the same network entity, as discussed in [25], and are referred to as “gene” during the remainder of the paragraph.

In order to generate a high-quality gene–gene network, each data layer is combined into a single layer network. This is achieved by retrieving gene–gene relationships based on common neighbors from the UKS. For each of the nine sources in Table S1, if two genes share at least one common association (such as, for example, are involved in the same pathway), they will have an edge in the resulting gene network.

Furthermore, the nine data layers are manually categorized into three sub-categories: Interaction, Regulation and Functional (Table S1). In order to create the flat multilayer network used in this study, the individual layers are merged hierarchically. All layers contained in a sub-category are merged into a binary network, where an edge in the merged network indicates its existence in at least one of the single layers. The three sub-category gene networks are then merged in a similar fashion into the final network, with the difference that only edges that are represented in all the three sub-categories are contained in the final network.

### 2.5. Characterization of Drugs Properties

In order to prioritize drugs potentially targeting nodes of the networks, we simultaneously evaluated multiple drug properties: (1) mechanism of action; (2) secondary structure; (3) the length of shortest path among drug targets and (4) drugs coverage on the multi-layer integrated network.

The paradigm that we follow in this study is to maximize the effectiveness of drug repositioning by prioritizing drug molecules which show the most dissimilar MOA, the most dissimilar secondary structure and the widest effect on the multi-layer network, expressed in terms of shortest path and coverage. The distances among MOA networks built on the L1000 profiles were calculated by computing a Hamming–Ipsen–Mikhailov metric (HIM), implemented in the nettools R package (https://github.com/filosi/nettools (accessed on 14 February 2018)). The MOA of drugs was inferred only for perturbational profiles for which more than five replicates were available in L1000.

Levenshtein pairwise distances among drug SMILES were then computed using the stringdist CRAN package [28].

Furthermore, shortest paths between the target genes of drugs in the cancer network were computed through the use of the igraph CRAN package. In the case of drugs having multiple target genes, the value associated with the shortest path was computed as the average length of the shortest paths connecting each possible pair of genes from the targets of the drugs under consideration.

Finally, we quantified the effect of each drug on the network in terms of both number of targets and their relevance within the cancer network by computing a score (effect score, ES), expressed by the following formula:

(2) $$\mathrm{ES}\;={\;N}_{t}\;÷M_{R}$$

where N_t_ is the number of targets for each drug; M_R_ is the median value of the rank indices of drug targets in the network, indicating the relevance of the drug targets within the cancer network in terms of centrality measures and SS.

### 2.6. Final Drug Prioritization

We defined a candidate drug combination as a subset x⊂Σ where Σ is the collection of all drugs considered in each experiment.

We also evaluated the criteria for each drug combination as follows:

(1). MOA: the drugs in x must have the most dissimilar mechanisms of action: $\max_{x\;\subset \;\Sigma }\;\mathrm{MOA}\left(x\right)$, where $\mathrm{MOA}\left(x\right)$ is the average HIM distance between each pair of drugs in x: $\mathrm{MOA}\left(x\right)=\frac{1}{\left|x\right|}\sum_{\left.{\left\{d\right.}_{I},{\;d}_{j}\right\}\subset \;x}^{}\mathrm{HIM}\left(d_{I},d_{j}\right)$, where $\left|x\right|$ is the number of drugs in x;
(2). SMILES: the drugs in x must have the most different secondary structure: $\max_{x\;\subset \;\Sigma }\;\mathrm{SMILES}\left(x\right)$, where $\mathrm{SMILES}\left(x\right)$ is the average Levenshtein distance between each pair of drugs in x: $\mathrm{SMILES}\left(x\right)=\frac{1}{\left|x\right|}\sum_{\left.{\left\{d\right.}_{I},{\;d}_{j}\right\}\subset \;x}^{}L\left(d_{I},d_{j}\right)$;
(3). TARGETS: the drugs in x must target genes which are as far as possible between themselves in the cancer network: $\max_{x\;\subset \;\Sigma }\;\mathrm{TARGETS}\left(x\right)$, where $\mathrm{TARGETS}\left(x\right)$ is the average length of the shortest paths between the sets of targets of each pair of drugs in x: $\mathrm{TARGETS}\left(x\right)=\frac{1}{\left|x\right|}\sum_{\left.{\left\{d\right.}_{I},{\;d}_{j}\right\}\subset \;x}^{}\mathrm{SP}\left(d_{I},d_{j}\right)$;
(4). COVERAGE: the drugs in x must target as many genes as possible in the cancer network: $\max_{x\;\subset \;\Sigma }\;\mathrm{ES}\left(x\right)$;
(5). SIZE: we want the smallest subset of drugs $\min_{x\subset \Sigma }\left|x\right|$.

Since we want to prioritize the drug combinations according to several different criteria, we framed the task as a multi-objective combinatorial optimization problem as follows:

(3) $$\max_{x\subset \Sigma }\left(\mathrm{MOA}\left(x\right),\;\mathrm{SMILES}\left(x\right),\;\mathrm{TARGETS}\left(x\right),\;\mathrm{COVERAGE}\left(x\right),-\mathrm{SIZE}\left(x\right)\right)$$

To solve this problem, we implemented a genetic algorithm strategy by using the DEAP computational framework [29], implemented in python.

In the proposed solution, a population of 100 individuals is considered. Each individual chromosome (denoted as c) is encoded as a binary vector of length equal to the number of drugs in Σ, $c\;\in \;{\left\{0,\;1\right\}}^{\left|\Sigma \right|}$. A 1 in the i-th position denotes the presence of the i-th drugs in the drug combination. Each bit position in the chromosome is randomly initialized to 1 with a probability of 30%.

For each iteration of the genetic algorithm, 20 new offspring are generated, by randomly applying the crossover and mutation operators with 70% and 30% of probability, respectively.

When crossover is chosen, two individuals of the population are randomly selected and split at a random position, and two offspring chromosomes are generated by mixing the left and right parts of the parent chromosomes. Only the first child is included in the set of offspring individuals and the other is discarded.

In case mutation is selected, each bit of the chromosome has a chance of 10% to be swapped with another bit in the same chromosome chosen at random. After 20 new individuals are generated, they are evaluated according to our criteria. These individuals, together with the current population, are ranked according to the NSGA-II strategy [30], and the top 100 individuals will form the population for the next iteration.

In addition, a Hall of Fame (HoF) mechanism was implemented. A list of the 10 best candidate solutions encountered during the execution of the algorithm is updated at the end of each iteration. Eventually, at the end of the execution, the HoF will contain the best individuals across the whole optimization process. Each solution in the HoF represents a different trade-off between the criteria along the Pareto front of the objective space.

## 3. Results and Discussion

In this study, we developed a network-based computational pipeline aimed at identifying candidate drugs to repurpose and finding custom-size sets of drugs that may be considered for combination therapy. This is fulfilled by integrating well established computational methods, such as gene co-expression network analysis, with drug properties, such as the mechanism of action, chemical structure and topological properties of the targets.

### 3.1. Implementation

The first step in any transcriptomics-based drug and drug-combination repositioning study, is the evaluation of the transcriptional deregulation characterizing the pathological condition. Our pipeline allows the characterization of the disease-related transcriptional alterations based on both altered gene expression levels with respect to the controls, and/or altered expression variability due to the pathological condition. In addition to differentially expressed genes between the diseased tissues and the healthy ones, genes showing a high expression variability across the collection of pathological tissues (and that might not show differential expression) might underline an impairment in their regulatory mechanisms. indicating a possible role in the expression of the pathological phenotype.

**Figure 1:**
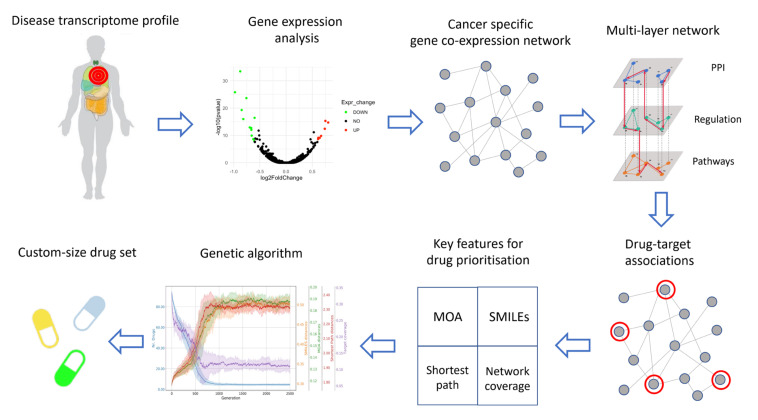
Computational workflow for the detection of drug combinations strategy for drug repositioning.

The second step of the pipeline consists of the inference of the disease network, based on the co-expression of the selected genes (Figure 1).

The inference is performed by using INfORM, which is an ensemble method that ensures robustness to the constructed network representing the molecular makeup of the disease under consideration. The integration of multiple complementary data sources enhances the performance of inference tasks [25,31]. For this reason, we integrated nine different resources, and in order to restrict the analysis to the biologically meaningful gene–gene connections of the disease network, we compiled integrated prior knowledge encompassing three complementary layers of information: (i) protein–protein interactions (PPI); (ii) regulatory interactions and (iii) interactions in functional pathways. Therefore, we selected edges having evidence of (1) connecting co-expressed genes in the disease network; (2) connecting two physically interacting protein products; (3) connecting two genes involved in a regulation process and (4) connecting two genes which are involved in the same pathway (Figure 1).

The drug–target association step represents the core of our hybrid pipeline, as both the functional- and structural-based approaches converge (Figure 1). We exploited the network representation of the disease molecular landscape in order to identify drugs with repurposing potential via inference of topological measures. The prediction of candidate drugs to repositioning, in fact, is strictly influenced by the location of their target within the disease network (see below). Moreover, a good evaluation of drug combinations is dependent on the multiple and integrated topological relationships among drug targets. For instance, if two drug targets are directly connected (or, in other words, are first degree neighbors), most probably these drugs may have similar mechanisms of action, represented by partially overlapping areas of genes on the network. This functional redundancy is not desirable when looking for combinations of drugs with complementary effects. Once a robust and biologically meaningful disease network is obtained, the following drug properties are considered in order to identify the most effective combination of drugs: (1) mechanism of action (MOA); (2) secondary structure; (3) drug targets’ shortest paths and (4) area of action of the drug on the disease network (Figure 1).

MOA: The proof-of-principle of considering the mechanism of action as a criterion for drug prioritization is that it is desirable to achieve combinations of drugs with the most different MOA, in order to minimize functional redundancy. In the present pipeline, the mechanism of action of drugs targeting genes in the disease network is represented by co-expression networks inferred by using the perturbational profiles of drug treatments on appropriate cell lines. Networks showing different patterns of connectivity will reflect drugs with different MOA and, hence, will be prioritized.

Secondary structure: Based on the widespread concept that the structure makes the function, drugs with a similar chemical structure are likely to have similar mechanisms of action. Therefore, as for the case of the MOA also for the chemical structure, it is desirable to prioritize drugs with the most different chemical structure. The Simplified Molecular Input Line Entry System (SMILES) is a common representation of a bidimensional model of chemical compounds, including drugs. Comparing the SMILES of different drugs is equivalent to comparing their chemical structure. Therefore, by computing the edit distance between drug SMILES, the distance between the chemical structures of drugs is considered. For the same principle as the MOA, the computed distance between SMILES is employed to prioritize drugs with different chemical structures.

Drug target shortest path: Another key feature of the drugs under consideration that is taken into account in this pipeline is their area of action on the disease network. In order to maximize the therapeutic effect of the drug combination, the action of drugs involved in a combination therapy should ideally be exerted on far, and ideally non-overlapping, areas of the disease network. To prioritize drugs showing such a property, we compute the distance between drug targets, by calculating the length of the shortest paths among their targets. The prioritized drugs show the longest shortest path in the disease network among their targets.

Drug area of action: For the same principle, the neighborhood of the drug targets is taken into consideration. Specifically, this pipeline prioritizes the drugs whose targets have the largest, non-overlapping neighborhoods.

Combination therapy offers multiple advantages compared to monotherapy, by providing higher efficacy with a lower individual dose, and therefore a safer toxicity profile. While identification of drug combinations with the lowest toxicity is often driven by serendipity in clinics, a rule of thumb consists in selecting the minimum number of compounds to combine in order to achieve the therapeutic effect. In order to systematically identify the smallest subset of drugs that would meet the desired criteria, the last step of our analysis results in a combinatorial multi-objective problem (Figure 1). Genetic algorithms are well established tools to solve this kind of problem. In particular, here we applied a multi-objective genetic strategy based on the NSGA-II selection algorithm, which is known to guarantee better computational complexity management and has a better convergence near to the true Pareto-optimal [32,33].

### 3.2. Case Study

Here we showcase the capabilities of our computational approach to identify candidate drug combinations by analyzing publicly available transcriptomics data from The Cancer Genome Atlas.

Based on the filtering criteria described in the Methods Section, we selected the following cancers as case studies: Invasive Breast Cancer (BRCA), Colon adenocarcinoma (COAD), Hepatocellular carcinoma (LIHC), Lung squamous cell carcinoma (LUSC) and Prostate adenocarcinoma (PRAD). The human primary cancers considered in this study and the relative number of patients (and paired samples) considered for this study are reported in Tables S2–S7. The drugs that have been tested in each of the considered cancer types are reported in Table S8. A comparison of the characteristics of currently available network-based and machine learning algorithms to derive drug combinations is reported in Table S9.

**Figure 2:**
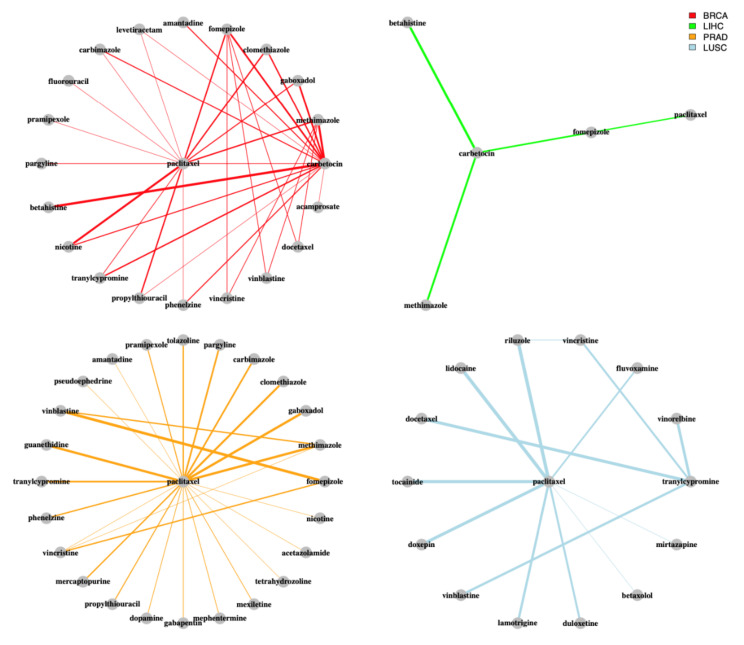
Network representation of the drug combinations in the considered cancers. The color of the edges indicates the cancer type. The thickness of the edges indicates the number of occurrences of the combination in the solutions of the genetic algorithm.

Our analysis highlights paclitaxel as the most frequent drug over all the obtained combinations (Figure 2).

Paclitaxel is known to target gene products belonging to the tubulin family and is used as a chemotherapeutic for several malignancies, including breast, esophageal, pancreatic and cervical cancer. Its role in cancer treatment is well assessed and included in the routine practice [34]. Altered cell motility is one of the hallmarks of metastatic cancer, which is responsible for the majority of cancer deaths. Furthermore, it has been proved that abnormal expression of cytoskeletal and cytoskeletal-associated proteins are responsible for chemoresistance [35].

In addition to paclitaxel, other cytostatics that target tubulin proteins were found in multiple drug combinations in BRCA, PRAD and LUSC, such as vinblastine or vincristine. Drug combinations in LIHC and COAD were, instead, centered around tanespimycin and navitoclax, respectively. As multiple trials have evaluated the effectiveness of a cytostatic together with a non-anticancer drug, our analysis suggests new potential combinations that have not been tested yet for breast and prostate cancer such as paclitaxel and clomethiazole [36,37,38,39,40,41]. The latter is a psychotropic drug used as a sedative targeting several subunits of the Gamma-Aminobutyric Acid receptors (Types A, B, D, G, among others). In prostate cancer, aberrant expression levels of these kinds of receptors have been previously reported to increase cellular proliferation [42] and to promote cellular invasiveness [43]. Although some in vitro studies have been carried out in order to assess the effectiveness of Gamma-Aminobutyric Acid receptors agonists or antagonists on cell proliferation [43,44,45], to the best of our knowledge, no in vitro tests have been carried out employing clomethiazole alone or in combination with other compounds in order to assess its antiproliferative action. For this reason, the anticancer effectiveness of clomethiazole needs further experimental verification. In BRCA, other drugs targeting the Gamma-Aminobutyric Acid receptors’ systems identified by our analysis pipeline include gaboxadol (in combination with both paclitaxel and carbetocin) and acamprosate. In colon adenocarcinoma, we found pentobarbital in combination with navitoclax as one of the top combinations. The anticancer efficacy of pentobarbital on animal models of colon adenocarcinoma was demonstrated in 2005 by Thaker and colleagues [46]. They demonstrated that the treatment with pentobarbital of mice affected by artificially induced colon adenocarcinoma (through HT29 cell line injection) reduced the tumor weight (primary tumor and liver metastases) in respect of the control group, while showing similar tumor incidence.

**Figure 3:**
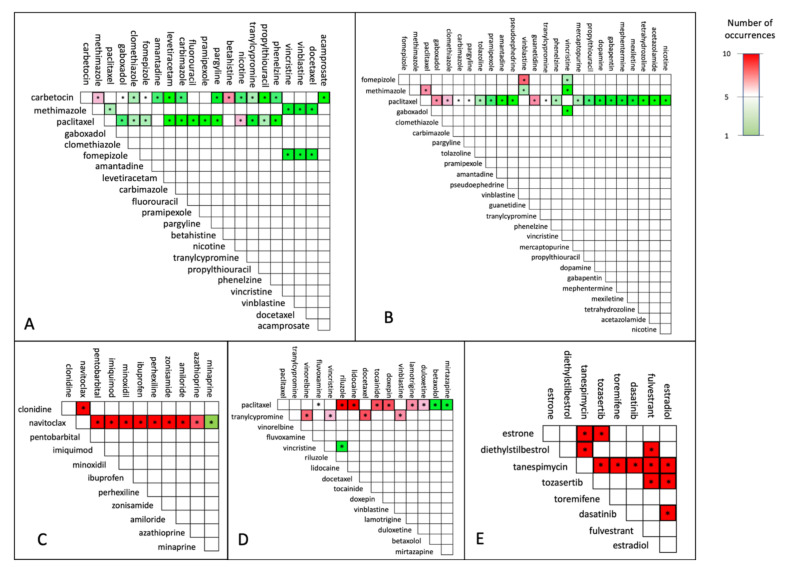
Overview of drug combinations obtained for the cancers under consideration. (**A**)—invasive breast cancer (BRCA); (**B**)—prostate adenocarcinoma (PRAD); (**C**)—colon adenocarcinoma (COAD); (**D**)—lung squamous cell carcinoma (LUSC); (**E**)—hepatocellular carcinoma best pairs (LIHC).

Although cytostatics are among the most effective approved group of drugs for cancer therapy so far, they come with a drawback which is their high toxicity, and acute as well as long-term adverse events. Thus, effective drug combinations that obviate the need for cytostatics are highly desirable. Overall, carbetocin is the second most recurrent drug in all the combinations obtained from our analysis, after paclitaxel. In BRCA, the most frequently represented combination is carbetocin and betahistine, which belong to the group of hormonals (Anatomical Therapeutical Chemical (ATC) code H) and nervous system drugs (ATC code N), respectively (Figure 3A).

Carbetocin is an oxytocin analogue that is mainly used to prevent postpartum hemorrhage. Several studies have shown its inhibitory action on breast tumor growth both in vitro and in vivo [47,48], in mouse, rat and human. Since several breast cancer cell lines show an increased expression of OXTR encoding the oxytocin receptor, oxytocin receptor-mediated signaling is gaining interest for its role in breast cancer development and progression. Thus, modulators of OTR-specific G protein-dependent or independent pathways need further investigation [48]. Betahistine is an antagonist of both histamine H3 receptor and histamine H1 receptor [49]. High histamine levels have been detected in both breast cancer patients and cell lines, and this was suggested to be directly correlated with malignancy [50,51,52,53,54]. Moreover, histamine levels were associated with cell differentiation and apoptosis in several breast cancer cell lines [52,55]. Importantly, some antihistaminergic drugs have been proved to reduce the cytokines’ levels and inflammation that characterizes the tumor microenvironment, which plays a decisive role in the tumor progression and therapy resistance [56,57].

Noteworthy, multiple drugs selected in combination with paclitaxel and vinblastine are indicated for central nervous system diseases. For instance, phenelzine and tranylcypromine were recurrently found in combination with paclitaxel and vinblastine in BRCA and PRAD, indicating that combining an anticancer drug with a monoamine oxidase (MAO) inhibitor can impact the transcriptional network of BRCA substantially [58]. As antidepressants are often used in the supportive care of cancer patients, the combination of anticancer drugs such as paclitaxel and antidepressants such as phenelzine or tranylcypromine, as suggested for both BRCA and PRAD by our analysis, might be one of the most straight-forward drug combinations to test in trials. In fact, establishing such trials might be coherent for the group of cancer patients developing comorbid depressive disorders.

Three combinations for PRAD suggest both paclitaxel and vinblastine together with drugs for the treatment of hyperthyroidism, such as methimazole and carbimazole. Of note, several completed clinical trials report successful outcomes for treatment of PRAD patients with a combination of cytostatics and hormonal drugs. On the other hand, in LIHC, the most frequent drug found in the combinations is tanespimycin, which is a derivative of the antibiotic geldanamycin and is currently employed in the treatment of several solid tumors [59] and multiple myeloma. The MOA of tanespimycin resembles the one of geldanamycin, by binding to the same molecular target HSP90, which consolidates the folding and stability of several intracellular messengers involved in cell growth, survival and development [60]. In COAD, all combinations contain navitoclax, which is an Bcl-2 antagonist and has been already tested in combination with other drugs for the treatment of solid tumors (clinicaltrials.gov -> “navitoclax, https://pubmed.ncbi.nlm.nih.gov/34575429/ (accessed on 11 October 2021)).

Our case study shed light on both commonalities and differences in the drug combination predictions among the considered cancer types. In BRCA, LUSC and PRAD the drug combinations are mainly composed by a drug targeting the tubulin system (such as paclitaxel, vinblastine, vincristine, docetaxel, vinorelbine) already employed in the clinical treatment of several cancers, and a drug belonging to an unusual functional category, such as hormonal (carbimazole, methimazole) and psychoanaleptics (phenelzine, tranylcypromine). In LIHC, the drug combinations are instead characterized by the quasi-ubiquitous presence of tanespimycin, belonging to the category of antitumor antibiotics in combination with aurora-kinase inhibitors and estrogens, such as estrone. Finally, our analysis revealed that in COAD navitoclax is present in all the combinations together with drugs belonging to a plethora of different functional categories, such as psycholeptics (pentobarbital), antidepressants, calcium channel blockers (perhexiline) and antihypertensives (clonidine).

Our results suggest combinations of different groups of drugs that have been already tested or approved for cancer therapy. The missing data on immunomodulatory drugs present a limitation for our drug repositioning and combination approach, as nowadays immunomodulatory drugs are often used for the treatment of tumors/cancers, also in combination with classical chemotherapeutics. Although our study does not add information for this particular type of drug combination, our in silico drug repurposing approach reveals new drug combinations with high impact on the tumor transcriptional networks.

Despite paclitaxel and carbetocin being the two most recurrent drugs in the obtained combinations, a combination involving both of these drugs was not obtained. Although the two drugs show a different mechanism of action in the cell lines under consideration, a different chemical structure and act on different targets, our predictions do not indicate the combination between paclitaxel and carbetocin as worthy of further investigation. On the other hand, while several drugs have been found in combination with only one among paclitaxel and carbetocin, only a few drugs have been indicated as suitable for repositioning and combination with both of them. For instance, in breast cancer, clomethiazole, gaboxadol, fomepizole, methimazole and tranylcypromine appear in combination with both of the drugs, while betahistine, carbimazole and amantadine are suggested to be suitable in combination only with paclitaxel.

### 3.3. Robustness and Stability Evaluation

We assessed the robustness of the genetic algorithm with respect to random initializations by running each use case ten times. As reported in Figure 3, the majority of drug combinations is stable across runs, implying that the genetic algorithm is almost insensitive to the initial random state of the population and able to converge to robust multi-objective optima.

**Figure 4:**
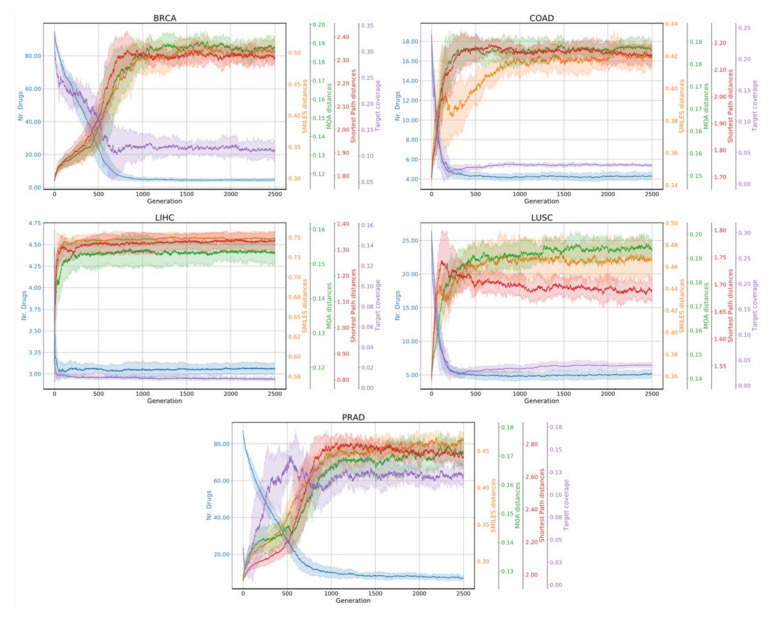
Trace plots showing the performance of the genetic algorithm in terms of stability of the obtained drug combinations. The color of the traces indicate the objective function that is optimized in the genetic algorithm.

We also report in Figure 4 the trace of each objective function for each use case during the evolution process across the ten runs.

In all cases the exploratory phase lasts for about 1000 generations (the use cases with fewer annotated drugs stabilize faster due to the reduced space of possible combinations), while the remainder of the generations is spent exploring the various trade-offs between each objective.

## 4. Conclusions

In this study, we built a computational framework to prioritize drug combinations by integrating mechanistic and chemocentric approaches. By identifying the minimum set of drugs which maximizes the therapeutic effect in a purely data-driven and systematic way, this pipeline is suitable to test and screen drug combinations with the highest efficacy and lowest toxicity in any complex disease. Finally, being based on transcriptional evidence and prior knowledge, this framework could aid developing therapeutic options for rare diseases, where identifying the pathobiological mechanism and further drug targets is still a practical and economic challenge. In order to prove the effectiveness of our framework, we performed a case study on five cancer types, including invasive breast cancer (BRCA), liver hepatocellular carcinoma (LIHC), prostate adenocarcinoma (PRAD), stomach adenocarcinoma (STAD), colon adenocarcinoma (COAD) and lung adenocarcinoma (LUAD). Our approach allowed the identification of relevant drug combinations, involving both drugs known to be effective against cancer progression and new compounds as candidates for repositioning in cancer therapy. Although the research of alternative drug molecular targets is still challenging, this study introduces a novel computational framework, the druggability map, as an instrument to guide the prioritization of drug combinations and repositioning based on integration of mechanistic characteristics of the disease and intrinsic properties of the drugs.

## Data Availability

The R code to run the pipeline presented in this study is available at https://github.com/antoniofederico87/drugMap (accessed on 24 February 2022).

## Supplementary Materials

The following supporting information can be downloaded at: https://www.mdpi.com/article/10.3390/cancers14082043/s1, Table S1: Sources used in the respective UKS layers to create the gene–gene network used in this study, together with their data types used and assigned data layers; Table S2: Overview of general network characteristics for each of the investigated cancers; Table S3: Results of the pipeline for invasive breast cancer (BRCA); Table S4: Results of the pipeline for liver hepatocellular carcinoma (LIHC); Table S5: Results of the pipeline for prostate adenocarcinoma (PRAD); Table S6: Results of the pipeline for colon adenocarcinoma (COAD); Table S7: Results of the pipeline for lung squamous cell carcinoma (LUSC). Table S8: Drugs considered in the analysis of the cancer types included in the case study. Table S9: Comparison of network-based and machine learning-based drug combination algorithms
