# Supplementary material for: Integrated Network Pharmacology Approach for Drug Combination Discovery: A Multi-Cancer Case Study
Source: Cancers (Basel). 2022 Apr 18;14(8):2043. doi: 10.3390/cancers14082043 (PMC9028433; doi:10.3390/cancers14082043)
Supplement: Supplementary file 1 [file cancers-14-02043-s001.zip › cancers-1632965-supplementary.pdf]

***Table S1**: Sources used in the respective UKS layers to create the gene - gene network used in this study, together with their data types used and assigned data layers.*

Source	Data Type	Data Layer	Website URL
STRING (Szklarczyk et al. 2019)	PPI	Interaction	https://string-db.org/
HIPPIE (Alanis-Lobato et al. 2017)	PPI	Interaction	http://cbdm-01.zdv.uni-mainz.de/~mschaefer/hippie/
HitPredict (Patil et al. 2011)	PPI	Interaction	http://www.hitpredict.org/
KEGG (Kanehisa et al. 2021)	PPI	Interaction	https://www.genome.jp/kegg/
Reactome (Jassal et al. 2020)	PPI	Interaction	https://reactome.org/
Ensembl (Howe et al. 2021)	Paralog Information	Interaction	https://www.ensembl.org/
Panther (Mi et al. 2021)	Protein Family Gene assignment	Interaction	http://www.pantherdb.org/
GO (Ashburner et al. 2000)	Cellular Component Gene association	Interaction	http://geneontology.org/
GO (Ashburner et al. 2000)	Biological Process Gene association	Functional	http://geneontology.org/
GO (Ashburner et al. 2000)	Molecular Function Gene association	Functional	http://geneontology.org/
TargetScan (McGeary et al. 2019)	mirRNA regulates Gene	Regulation	http://www.targetscan.org/
TRRUST (Han et al. 2018)	Gene regulates Gene	Regulation	https://www.grnpedia.org/trrust/
JASPAR (Fornes et al. 2020)	Gene regulates Gene	Regulation	https://jaspar.genereg.net/
KEGG (Kanehisa	Gene Pathway	Functional	

et al. 2021)	associations		https://www.genome.jp/kegg/
Reactome (Jassal et al. 2020)	Gene Pathway associations	Functional	https://reactome.org/
WikiPathways (Martens et al. 2021)	Gene Pathway associations	Functional	https://www.wikipathways.org/

***Table S2**: Overview of general network characteristics for each of the investigated cancers.*

Cancer type	number of paired cancer/normal samples	number of genes - edges in the cancer network	number of genes - edges in the prior network	number of drugs investigated in this study
Invasive Breast Cancer (BRCA)	112 - 224	4,568 -1,441,146	2,566 - 242,226	316
Colon adenocarcinoma (COAD)	41 - 82	4,416 - 1,111,603	2,254 - 190,091	62
Hepatocellular carcinoma (LIHC)	50 - 100	3,965 - 715,499	2,369 - 133,105	11
Lung squamous cell carcinoma (LUSC)	49 - 98	4035 - 2,795,288	2,648 - 426,679	87
Prostate adenocarcinoma (PRAD)	52 - 104	4,477 - 681,517	2,131 - 119,770	294

Table S3 *- Results of the pipeline for invasive breast cancer (BRCA). In each row are indicated the drugs in the combination, the frequency of occurrence in the output of the genetic algorithm, their targets, the respective disease network rank and the area of action in terms of number of genes.*

Drug 1	Drug 2	Occurrence in GA solutions	Target genes 1	Target genes 2	BRCA network rank 1	BRCA network rank 2	Estimated area of action (nodes)
carbetocin	betahistine	7	OXTR	HRH3	1199	2322	283
methimazole	carbetocin	6	TPO	OXTR	906	1199	436
paclitaxel	nicotine	6	TUBA1C;TUBB4A;TUBB2B;TUBA3C;TUBA3E;TUBB3	CHRNA4	350;1269;1403;1837;2106;2304	2417	713
gaboxadol	carbetocin	5	GABRA3;GABRA2;GABRA5;GABRA4	OXTR	1081;1586;1934;1965	1199	623
carbetocin	fomepizole	5	OXTR	ADH1C;ADH1A;ADH1B	1199	256;908;947	843
clomethiazole	paclitaxel	4	GABRA3;GABRA4;GABRA2;GABRG2;GABRA5;GABRD;GABRQ;GABRE;GABRG1	TUBA1C;TUBB4A;TUBB2B;TUBA3C;TUBA3E;TUBB3	802;1023;1081;1388;1586;1749;1934;1965;2474	350;1269;1403;1837;2106;2304	1128
carbetocin	tranylcypromine	4	OXTR	MAOA	1199	969	565
fomepizole	paclitaxel	4	ADH1C;ADH1A;ADH1B	TUBA1C;TUBB4A;TUBB2B;TUBA3C;TUBA3E;TUBB3	256;908;947	350;1269;1403;1837;2106;2304	1137
methimazole	paclitaxel	4	TPO	TUBA1C;TUBB4A;TUBB2B;TUBA3C;TUBA3E;TUBB3	906	350;1269;1403;1837;2106;2304	889
paclitaxel	propylthiouracil	4	TUBA1C;TUBB4A;TUBB2B;TUBA3C;TUBA3E;TUBB3	TPO;DIO1	350;1269;1403;1837;2106;2304	906;1755	919
carbetocin	clomethiazole	4	OXTR	GABRA3;GABRA4;GABRA2;GABRG2;GABRA5;GABRD;GABRQ;GABRE;GABRG1	1199	802;1023;1081;1388;1586;1749;1934;1965;2474	766
carbetocin	carbimazole	3	OXTR	TPO	1199	906	436
amantadine	carbetocin	3	GRIN2D;GRIN2B	OXTR	386;1486	1199	632
gaboxadol	paclitaxel	3	GABRA3;GABRA2;GABRA5;GABRA4	TUBA1C;TUBB4A;TUBB2B;TUBA3C;TUBA3E;TUBB3	1081;1586;1934;1965	350;1269;1403;1837;2106;2304	1007
carbetocin	nicotine	3	OXTR	CHRNA4	1199	2417	256
carbetocin	phenelzine	3	OXTR	MAOA	1199	969	565

paclitaxel	tranylcypromine	2	TUBA1C;TUBB4A;TUBB2B;TUBA3C;TUBA3E;TUBB3	MAOA	350;1269;1403;1837;2106;2304	969	946
methimazole	vincristine	2	TPO	TUBB3;TUBA3E;TUBA3C;TUBB2B;TUBB4A;TUBA1C	906	350;1269;1403;1837;2106;2304	889
carbetocin	pargyline	2	OXTR	MAOA	1199	969	565
fomepizole	vinblastine	2	ADH1C;ADH1A;ADH1B	TUBA3C;TUBB4A;TUBA3E;TUBA1C;TUBB2B;TUBB3	256;908;947	350;1269;1403;1837;2106;2304	1137
methimazole	vinblastine	2	TPO	TUBA3C;TUBB4A;TUBA3E;TUBA1C;TUBB2B;TUBB3	906	350;1269;1403;1837;2106;2304	889
fomepizole	vincristine	2	ADH1C;ADH1A;ADH1B	TUBB3;TUBA3E;TUBA3C;TUBB2B;TUBB4A;TUBA1C	256;908;947	350;1269;1403;1837;2106;2304	1137
fomepizole	docetaxel	2	ADH1C;ADH1A;ADH1B	TUBA1C;TUBB2B;TUBA3E;TUBB4A;TUBA3C;TUBB3	256;908;947	350;1269;1403;1837;2106;2304	1137
methimazole	docetaxel	2	TPO	TUBA1C;TUBB2B;TUBA3E;TUBB4A;TUBA3C;TUBB3	906	350;1269;1403;1837;2106;2304	889
levetiracetam	carbetocin	1	CACNA1B	OXTR	1627	1199	298
carbetocin	propylthiouracil	1	OXTR	TPO;DIO1	1199	906;1755	472
paclitaxel	phenelzine	1	TUBA1C;TUBB4A;TUBB2B;TUBA3C;TUBA3E;TUBB3	MAOA	350;1269;1403;1837;2106;2304	969	946
carbimazole	paclitaxel	1	TPO	TUBA1C;TUBB4A;TUBB2B;TUBA3C;TUBA3E;TUBB3	906	350;1269;1403;1837;2106;2304	889
levetiracetam	paclitaxel	1	CACNA1B	TUBA1C;TUBB4A;TUBB2B;TUBA3C;TUBA3E;TUBB3	1627	350;1269;1403;1837;2106;2304	736
fluorouracil	paclitaxel	1	TYMS	TUBA1C;TUBB4A;TUBB2B;TUBA3C;TUBA3E;TUBB3	1385	350;1269;1403;1837;2106;2304	787
pramipexole	paclitaxel	1	DRD2	TUBA1C;TUBB4A;TUBB2B;TUBA3C;TUBA3E;TUBB3	1221	350;1269;1403;1837;2106;2304	878
pargyline	paclitaxel	1	MAOA	TUBA1C;TUBB4A;TUBB2B;TUBA3C;TUBA3E;TUBB3	969	350;1269;1403;1837;2106;2304	946
carbetocin	acamprosate	1	OXTR	GABRD;GABRQ;GRIN2B;GABRE;GABRA2;GRIN2D;GABRA3;GAB	1199	386;802;1023;1081;1388;1486;1586;1749;1934;1965;2474	938

				RA5;GABRA4;GABRG1;GABRG2			

Table S4 *- Results of the pipeline for liver hepatocellular carcinoma (LIHC). In each row are indicated the drugs in the combination, the frequency of occurrence in the output of the genetic algorithm, their targets, the respective disease network rank and the area of action in terms of number of genes.*

Drug 1	Drug 2	Drug 3	Occurrence in GA solutions	Target genes 1	Target genes 2	Target genes 3	LIHC network rank 1	LIHC network rank 2	LIHC network rank 3	Estimated area of action (nodes)
estrone	tanespimycin	NA	10	ESR1	HSP90AB1		203	175		875
diethylstilbestrol	tanespimycin	NA	10	ESR1	HSP90AB1		203	175		875
tanespimycin	tozasertib	estradiol	10	HSP90AB1	AURKA;AURKB	ESR1	175	61;305	203	1055
estrone	tozasertib	tanespimycin	10	ESR1	AURKA;AURKB	HSP90AB1	203	61;305	175	1055
tozasertib	tanespimycin	NA	10	AURKA;AURKB	HSP90AB1		61;305	175		897
tanespimycin	dasatinib	estradiol	10	HSP90AB1	EPHA2;PDGFRB	ESR1	175	1092;1113	203	1006
diethylstilbestrol	fulvestrant	tanespimycin	10	ESR1	ESR1	HSP90AB1	203	203	175	875
toremifene	tanespimycin	NA	10	ESR1	HSP90AB1		203	175		875
tozasertib	fulvestrant	tanespimycin	10	AURKA;AURKB	ESR1	HSP90AB1	61;305	203	175	1055

Table S5 *- Results of the pipeline for prostate adenocarcinoma (PRAD). In each row are indicated the drugs in the combination, the frequency of occurrence in the output of the genetic algorithm, their targets, the respective disease network rank and the area of action in terms of number of genes.*

Drug 1	Drug 2	Occurrence in GA solutions	Target genes 1	Target genes 2	PRAD network rank 1	PRAD network rank 2	Estimated area of action (nodes)
fomepizole	vinblastine	8	ADH1B	TUBA1A;TUBB4A;TUBA4A;TUBB2A;TUBB6	796	58;703;781;1294;1722	791

methimazole	paclitaxel	7	TPO	TUBA1A;TUBB6;TUBB2A;TUBB4A;TUBA4A	818	58;703;781;1294;1722	740
paclitaxel	guanethidine	7	TUBA1A;TUBB6;TUBB2A;TUBB4A;TUBA4A	SLC6A2	58;703;781;1294;1722	1606	740
gaboxadol	paclitaxel	7	GABRA1;GABRB3	TUBA1A;TUBB6;TUBB2A;TUBB4A;TUBA4A	1352;1927	58;703;781;1294;1722	767
clomethiazole	paclitaxel	6	GABRA1;GABRB3	TUBA1A;TUBB6;TUBB2A;TUBB4A;TUBA4A	1352;1927	58;703;781;1294;1722	767
carbimazole	paclitaxel	5	TPO	TUBA1A;TUBB6;TUBB2A;TUBB4A;TUBA4A	818	58;703;781;1294;1722	740
paclitaxel	tranylcypromine	5	TUBA1A;TUBB6;TUBB2A;TUBB4A;TUBA4A	MAOB	58;703;781;1294;1722	923	766
pargyline	paclitaxel	5	MAOB	TUBA1A;TUBB6;TUBB2A;TUBB4A;TUBA4A	923	58;703;781;1294;1722	766
tolazoline	paclitaxel	4	ADRA2B;ADRA1B;ADRA1D;ADRA1A	TUBA1A;TUBB6;TUBB2A;TUBB4A;TUBA4A	692;741;1036;1393	58;703;781;1294;1722	853
paclitaxel	phenelzine	4	TUBA1A;TUBB6;TUBB2A;TUBB4A;TUBA4A	MAOB	58;703;781;1294;1722	923	766
fomepizole	vincristine	4	ADH1B	TUBA4A;TUBB4A;TUBB2A;TUBA1A;TUBB6	796	58;703;781;1294;1722	791
paclitaxel	mercaptopurine	4	TUBA1A;TUBB6;TUBB2A;TUBB4A;TUBA4A	PPAT	58;703;781;1294;1722	870	724
methimazole	vinblastine	4	TPO	TUBA1A;TUBB4A;TUBA4A;TUBB2A;TUBB6	818	58;703;781;1294;1722	740
paclitaxel	propylthiouracil	3	TUBA1A;TUBB6;TUBB2A;TUBB4A;TUBA4A	TPO;DIO1	58;703;781;1294;1722	818;1832	749
pramipexole	paclitaxel	3	DRD2	TUBA1A;TUBB6;TUBB2A;TUBB4A;TUBA4A	834	58;703;781;1294;1722	772
paclitaxel	dopamine	2	TUBA1A;TUBB6;TUBB2A;TUBB4A;TUBA4A	ADRB1	58;703;781;1294;1722	250	786
paclitaxel	gabapentin	2	TUBA1A;TUBB6;TUBB2A;TUBB4A;TUBA4A	CACNB1;CACNA2D3;CACNA1D	58;703;781;1294;1722	387;973;980	913
paclitaxel	mephentermine	2	TUBA1A;TUBB6;TUBB2A;TUBB4A;TUBA4A	ADRA2B;ADRA1B;ADRA1D;ADRA1A;ADRB1;ADRB3	58;703;781;1294;1722	250;692;741;965;1036;1393	926
paclitaxel	mexiletine	2	TUBA1A;TUBB6;TUBB2A;TUBB4A;TUBA4A	SCN5A;SCN1A;SCN11A	58;703;781;1294;1722	361;1756;2045	915
amantadine	paclitaxel	1	GRIN3A	TUBA1A;TUBB6;TUBB2A;TUBB4	1799	58;703;781;1294;1722	729

				A;TUBA4A			
pseudoephedrine	paclitaxel	1	SLC6A2	TUBA1A;TUBB6;TUBB2A;TUBB4A;TUBA4A	1606	58;703;781;1294;1722	740
gaboxadol	vincristine	1	GABRA1;GABRB3	TUBA4A;TUBB4A;TUBB2A;TUBA1A;TUBB6	1352;1927	58;703;781;1294;1722	767
paclitaxel	tetrahydrozoline	1	TUBA1A;TUBB6;TUBB2A;TUBB4A;TUBA4A	ADRA1D;ADRA1B;ADRA2B;ADRA1A	58;703;781;1294;1722	692;741;1036;1393	853
paclitaxel	acetazolamide	1	TUBA1A;TUBB6;TUBB2A;TUBB4A;TUBA4A	CA4;CA12;CA2	58;703;781;1294;1722	1505;1536;1611	864
methimazole	vincristine	1	TPO	TUBA4A;TUBB4A;TUBB2A;TUBA1A;TUBB6	818	58;703;781;1294;1722	740
paclitaxel	nicotine	1	TUBA1A;TUBB6;TUBB2A;TUBB4A;TUBA4A	CHRNA4	58;703;781;1294;1722	1597	768

***Table S6** - Results of the pipeline for colon adenocarcinoma (COAD). In each row are indicated the drugs in the combination, the frequency of occurrence in the output of the genetic algorithm, their targets, the respective disease network rank and the area of action in terms of number of genes.*

Drug 1	Drug 2	Occurrence in GA solutions	Target genes 1	Target genes 2	COAD network rank 1	COAD network rank 2	Estimated area of action (nodes)
clonidine	navitoclax	10	ADRA2C	BCL2	1603	466	633
navitoclax	pentobarbital	10	BCL2	GABRA6;GABRP;GABRE;GABRG1;GABRD;GABRG2	466	881;993;1135;1253;1951;2211	952
navitoclax	imiquimod	10	BCL2	TLR7	466	582	764
navitoclax	minoxidil	10	BCL2	KCNJ11	466	1782	650
navitoclax	ibuprofen	10	BCL2	PTGS1	466	1447	707
navitoclax	perhexiline	10	BCL2	CPT2	466	129	656
navitoclax	zonisamide	10	BCL2	SCN9A;SCN11A;SCN7A;SCN3A	466	13;281;710;1403	1015
navitoclax	amiloride	10	BCL2	SCNN1B;SCNN1G	466	522;1566	831
navitoclax	azathioprine	9	BCL2	PPAT	466	138	606
navitoclax	minaprine	1	BCL2	DRD2;MAOA	466	133;1818	855

Table S7 *- Results of the pipeline for lung squamous cell carcinoma (LUSC). In each row are indicated the drugs in the combination, the frequency of occurrence in the output of the genetic algorithm, their targets, the respective disease network rank and the area of action in terms of number of genes.*

Drug 1	Drug 2	Occurrence in GA solutions	Target genes 1	Target genes 2	LUSC network rank 1	LUSC network rank 2	Estimated area of action (nodes)
paclitaxel	riluzole	10	TUBB1;TUBB4A;TUBB2B;TUBA3C;TUBA3E;TUBB3	SCN5A;SCN7A;SCN1A;SCN8A	1154;1242;1786;1974;2559;2591	357;682;1181;1725	1563
paclitaxel	lidocaine	10	TUBB1;TUBB4A;TUBB2B;TUBA3C;TUBA3E;TUBB3	SCN8A;SCN5A;SCN1A;SCN7A	1154;1242;1786;1974;2559;2591	357;682;1181;1725	1563
tranylcypromine	docetaxel	9	MAOB	TUBB2B;TUBA3E;TUBB4A;TUBB1;TUBA3C;TUBB3	1101	1154;1242;1786;1974;2559;2591	1172
paclitaxel	tocainide	9	TUBB1;TUBB4A;TUBB2B;TUBA3C;TUBA3E;TUBB3	SCN5A;SCN1A;SCN7A;SCN8A	1154;1242;1786;1974;2559;2591	357;682;1181;1725	1563
paclitaxel	doxepin	9	TUBB1;TUBB4A;TUBB2B;TUBA3C;TUBA3E;TUBB3	SLC6A2	1154;1242;1786;1974;2559;2591	1841	1101
vinorelbine	tranylcypromine	8	TUBB2B;TUBB1;TUBB4A;TUBA3C;TUBB3;TUBA3E	MAOB	1154;1242;1786;1974;2559;2591	1101	1172
tranylcypromine	vinblastine	7	MAOB	TUBA3C;TUBB4A;TUBA3E;TUBB1;TUBB2B;TUBB3	1101	1154;1242;1786;1974;2559;2591	1172
paclitaxel	lamotrigine	7	TUBB1;TUBB4A;TUBB2B;TUBA3C;TUBA3E;TUBB3	SCN7A;SCN5A;SCN1A;SCN8A	1154;1242;1786;1974;2559;2591	357;682;1181;1725	1563
paclitaxel	duloxetine	6	TUBB1;TUBB4A;TUBB2B;TUBA3C;TUBA3E;TUBB3	SLC6A2;SLC6A4	1154;1242;1786;1974;2559;2591	1417;1841	1279
tranylcypromine	vincristine	6	MAOB	TUBB3;TUBB1;TUBA3E;TUBA3C;TUBB2B;TUBB4A	1101	1154;1242;1786;1974;2559;2591	1172
fluvoxamine	paclitaxel	5	SLC6A4	TUBB1;TUBB4A;TUBB2B;TUBA3C;TUBA3E;TUBB3	1417	1154;1242;1786;1974;2559;2591	1190
vincristine	riluzole	2	TUBB3;TUBB1;TUBA3E;TUBA3C;TUBB2B;TUBB4A	SCN5A;SCN7A;SCN1A;SCN8A	1154;1242;1786;1974;2559;2591	357;682;1181;1725	1563
paclitaxel	betaxolol	1	TUBB1;TUBB4A;	ADRB1	1154;1242;1786	1312	1192

			TUBB2B;TUBA3C;TUBA3E;TUBB3		;1974;2559;2591		
paclitaxel	mirtazapine	1	TUBB1;TUBB4A;TUBB2B;TUBA3C;TUBA3E;TUBB3	HTR2C	1154;1242;1786;1974;2559;2591	1197	1203

Table S8 *– Drugs considered in the analysis of the cancer types included in the case study. The cancer cell lines from which the drug sensitivity profiles have been derived are: MCF7 for BRCA, HEPG2 for LIHC, PC3 for PRAD, HT29 for COAD and A549 for LUSC.*

**Drugs**	**BRCA**	**COAD**	**LIHC**	**LUSC**	**PRAD**
acamprosate	x				
acetazolamide	x				x
acitretin	x				x
afatinib	x	x		x	
alectinib	x	x			
alfuzosin	x			x	x
alisertib	x				x
alitretinoin	x			x	x
alvocidib	x			x	
amantadine	x				x
ambrisentan	x				x
aminoglutethimide	x				x
amisulpride	x			x	x
amitriptyline	x				x
amlodipine	x			x	x
amoxapine	x				x
amsacrine	x				x
amuvatinib	x	x			x
anagrelide	x				x
aprepitant	x				
aripiprazole	x				x
articaine	x				x
asenapine	x				x
balsalazide	x				x

beclomethasone-dipropionate	x				
benperidol	x				x
benzbromarone	x	x			
bepridil	x				x
betahistine	x				
betamethasone-acetate	x				
betamethasone	x				
bezafibrate	x				
bimatoprost	x				
bosentan	x				x
brimonidine	x				x
brivanib	x	x		x	x
bromocriptine	x	x			x
budesonide	x	x			
bumetanide	x				
buparlisib	x	x			
bupivacaine	x				
cabergoline	x				x
cabozantinib	x	x		x	
canertinib	x	x		x	
capecitabine	x				
carbamazepine	x				x
carbenoxolone	x				
carbetocin	x				
carbimazole	x				x
carteolol	x				x
carvedilol	x				x
cediranib	x				x
chlorpromazine	x				x
chlorprothixene	x				x
cilastatin	x				
cinnarizine	x				x
ciprofibrate	x				
citalopram	x			x	
clebopride	x				x
clenbuterol	x				

clofarabine	x				x
clofibrate	x				
clomethiazole	x				x
clomipramine	x				
clonidine	x	x			x
clozapine	x				x
colchicine	x				x
cortisone-acetate	x				
crizotinib	x	x			
cyclobenzaprine	x				
cytarabine	x				
dacomitinib	x	x			x
dantrolene	x				x
danusertib	x				x
dasatinib	x	x	x	x	x
daunorubicin	x			x	x
desoximetasone	x				
dexamethasone-acetate	x				
dexamethasone	x	x			
dexfenfluramine	x				x
diazepam	x				x
digitoxin	x				x
digoxin	x				x
dihydroergotamine	x	x			
diltiazem	x			x	x
dinaciclib	x				
dinoprostone	x				
dipyridamole	x				x
disopyramide	x				x
disulfiram	x				x
dobutamine	x				x
docetaxel	x			x	x
dovitinib	x			x	x
doxapram	x				x
doxazosin	x				x
doxorubicin	x			x	x

doxycycline	x				
droperidol	x				x
duloxetine	x			x	x
eplerenone	x				
erlotinib	x				
escitalopram	x			x	
esmolol	x				x
ethotoin	x				x
etomidate	x				x
etoposide	x			x	x
felbamate	x			x	x
felodipine	x				x
fenofibrate	x				
fenoterol	x				
flavoxate	x				x
flecainide	x				x
floxuridine	x				
fludrocortisone-acetate	x				
flumazenil	x			x	x
flunisolide	x				
fluocinolone-acetonide	x	x			
fluocinonide	x				
fluorometholone	x	x			
fluorouracil	x				
fluoxetine	x			x	
fluphenazine	x	x			x
fluticasone-propionate	x				
fluvoxamine	x			x	
fomepizole	x				x
foretinib	x	x			x
formoterol	x				
fulvestrant	x		x		
furosemide	x				
gabapentin	x				x
gaboxadol	x				x
gefitinib	x				

gemcitabine	x				x
gemfibrozil	x				
guanabenz	x				x
guanfacine	x				x
haloperidol	x	x			x
hydrocortisone-hemisuccinate	x				
hydrocortisone-valerate	x				
hydrocortisone	x				
idarubicin	x			x	
imatinib	x	x			x
imipramine	x				x
isocarboxazid	x				x
isradipine	x			x	x
labetalol	x				x
lamotrigine	x			x	x
lapatinib	x				
latanoprost	x				
lestaurtinib	x				
levetiracetam	x				
levobunolol	x				x
levomepromazine	x				x
levothyroxine	x				
lidocaine	x			x	x
linifanib	x			x	
liothyronine	x				
lorazepam	x				x
loxapine	x			x	x
masitinib	x	x			x
mecamylamine	x				
medrysone	x				
memantine	x				x
mephentermine	x				x
mephenytoin	x				x
mepivacaine	x				x
mesoridazine	x				x
methazolamide	x				x

methimazole	x				x
methyldopa	x				x
methylprednisolone	x				
methysergide	x			x	
metoclopramide	x				x
mexiletine	x				x
mibefradil	x				
midodrine	x				x
midostaurin	x				x
mifepristone	x				x
milnacipran	x				x
minaprine	x	x			x
minoxidil	x	x			
mirtazapine	x			x	x
mitotane	x				x
mitoxantrone	x		x	x	x
molindone	x	x			x
motesanib	x			x	x
moxisylyte	x				x
nadolol	x				x
nalbuphine	x				
naloxone	x				
naltrexone	x				
naphazoline	x				x
nefazodone	x			x	x
neratinib	x	x		x	x
nialamide	x				x
nicardipine	x			x	x
nicotine	x				x
nifedipine	x			x	x
nimodipine	x	x		x	x
nintedanib	x	x			x
nisoldipine	x				x
nomifensine	x				x
norepinephrine	x				x
nortriptyline	x				x

olanzapine	x				x
olaparib	x				x
orantinib	x				x
orlistat	x				x
orphenadrine	x				x
osimertinib	x	x		x	
oxcarbazepine	x				x
oxprenolol	x				x
oxymetazoline	x				x
paclitaxel	x			x	x
pargyline	x				x
paroxetine	x			x	
pazopanib	x	x		x	x
pelitinib	x				
pentobarbital	x	x			x
pentoxifylline	x				x
pergolide	x				x
perphenazine	x				x
phenazopyridine	x				x
phenelzine	x				x
phenoxybenzamine	x				x
phensuximide	x				
phentolamine	x				x
phenytoin	x				x
pimozide	x	x			x
pinacidil	x				
pindolol	x				x
pioglitazone	x				
pramipexole	x				x
prazosin	x				x
prednisolone-acetate	x				
prednisolone	x				
prednisone	x				
prilocaine	x				x
primidone	x				x
procainamide	x				x

prochlorperazine	x				x
proglumide	x				x
promazine	x				x
propafenone	x				x
propofol	x				x
propranolol	x				x
propylthiouracil	x				x
protriptyline	x				x
quetiapine	x				x
quinidine	x			x	x
quizartinib	x	x		x	x
raloxifene	x				
raltitrexed	x			x	
ranolazine	x			x	x
regorafenib	x	x		x	x
riluzole	x			x	x
rimexolone	x				
risperidone	x			x	x
ritodrine	x	x		x	
rivaroxaban	x				
rizatriptan	x				
ropinirole	x				x
rosiglitazone	x				
rucaparib	x				x
salmeterol	x			x	
semaxanib	x				x
sertindole	x				x
sertraline	x			x	
sibutramine	x			x	x
sorafenib	x	x		x	x
sotalol	x	x		x	x
spironolactone	x				
sulfinpyrazone	x				
suloctidil	x				x
sumatriptan	x				
sunitinib	x			x	x

tandutinib	x				x
teniposide	x				x
terazosin	x				x
terbutaline	x				
tetracaine	x				x
tetrahydrozoline	x				x
theophylline	x				x
thioridazine	x	x		x	x
thiothixene	x				x
ticlopidine	x				
timolol	x				x
tivantinib	x	x			
tizanidine	x				x
tocainide	x			x	x
tolazoline	x				x
topiramate	x				x
toremifene	x		x		
tosedostat	x				
tozasertib	x		x	x	x
tranylcypromine	x	x		x	x
trazodone	x			x	
triamcinolone-acetonide	x	x			
triamcinolone	x	x			
trifluoperazine	x	x		x	x
triflupromazine	x				x
trimipramine	x				x
troglitazone	x				
vandetanib	x	x		x	x
varenicline	x				x
veliparib	x				x
venlafaxine	x				x
verapamil	x			x	x
vinblastine	x			x	x
vincristine	x			x	x
vinorelbine	x			x	x
zaleplon	x				x

ziprasidone	x				x
zolmitriptan	x				
zolpidem	x				x
zonisamide	x	x			x
amiloride		x			
azathioprine		x			x
cisapride		x			
enzastaurin		x			x
ibuprofen		x			x
imiquimod		x			
indoprofen		x			x
letrozole		x			x
navitoclax		x			
norethindrone		x			x
ondansetron		x		x	
palbociclib		x			
perhexiline		x			
progesterone		x		x	x
repaglinide		x			
tolazamide		x			
diethylstilbestrol			x		
entinostat			x		
estradiol			x		
estrone			x		
tamoxifen			x		
tanespimycin			x		
benazepril				x	
betaxolol				x	x
biperiden				x	
bupropion				x	x
doxepin				x	x
ibrutinib				x	
levonorgestrel				x	x
losartan				x	
maprotiline				x	x
omeprazole				x	x

ramipril				x	
scopolamine				x	
sildenafil				x	
telmisartan				x	
thalidomide				x	
tivozanib				x	
valsartan				x	
vardenafil				x	
abiraterone					x
acarbose					x
acebutolol					x
aliskiren					x
aminosalicylic-acid					x
amlexanox					x
anastrozole					x
aspirin					x
atomoxetine					x
benzydamine					x
bisoprolol					x
brinzolamide					x
bromfenac					x
celecoxib					x
chlordiazepoxide					x
danazol					x
desipramine					x
dexketoprofen					x
diazoxide					x
dibenzepin					x
diclofenac					x
diflunisal					x
dopamine					x
dorzolamide					x
dydrogesterone					x
etazolate					x
etodolac					x
etoricoxib					x

exemestane					x
finasteride					x
glafenine					x
guanethidine					x
iloperidone					x
isotretinoin					x
ketoprofen					x
ketorolac					x
lansoprazole					x
meclofenamic-acid					x
mefenamic-acid					x
megestrol-acetate					x
meloxicam					x
mercaptopurine					x
metoprolol					x
miglitol					x
modafinil					x
mycophenolate-mofetil					x
mycophenolic-acid					x
nabumetone					x
naproxen					x
norgestimate					x
norgestrel					x
oxaprozin					x
pantoprazole					x
phentermine					x
phenylbutazone					x
piroxicam					x
plinabulin					x
probenecid					x
procaine					x
pseudoephedrine					x
rabeprazole					x
reboxetine					x
rofecoxib					x
ropivacaine					x

safinamide					x
selegiline					x
sulfasalazine					x
sulindac					x
suprofen					x
tacrine					x
tolmetin					x
tretinoin					x
valdecoxib					x
vecuronium					x

Table S9 *– Comparison of network-based and machine learning-based drug combination algorithms.*

Category	***Requires experimental data***	***Approach***	***Validation***	***Reference***
Network based	*No*	*Overlapping drug-disease-drug modules in PPI network*	*Computational*	*PMID: 30867426*
Network based	*No*	*Highest enrichment of target pathways associated to disease*	*Computational/experimental*	*PMID: 34151561*
Network based	*No*	*Combination of topological and phenotypic properties of combination candidates in PPI network*	*Computational/experimental*	*PMID: 21689469*
SVM	*Yes*	*Predictive model based on single and combinatorial experimental data*	*Computational/experimental*	*PMID: 28085880*
Gradient boosting	*Yes*	*Predictive model based on the extraction of features from*	*Computational*	*PMID: 31338106*

		combinations of drugs		
Multiple ML models	*Yes*	*Predictive model based on the extraction of features from combinations of drugs*	*Computational*	*PMID: 30304987*
